# Genetic variation of *Picea abies* in response to the artificial inoculation of *Heterobasidion parviporum*

**DOI:** 10.1007/s10342-023-01534-3

**Published:** 2023-01-27

**Authors:** Blessing Durodola, Kathrin Blumenstein, Eeva Terhonen

**Affiliations:** 1 Department of Forest Botany and Tree Physiology, Faculty of Forest Sciences and Forest Ecology, Forest Pathology Research Group, Büsgen-Institute, University of Göttingen, Büsgenweg 2, 37077 Göttingen, Germany; 2 Forest Genetics and Forest Tree Breeding, Büsgen-Institute, Georg-August University Göttingen, Büsgenweg 2, 37077 Göttingen, Germany; 3 Chair of Pathology of Trees, Faculty of Environment and Natural Resources, Institute of Forestry, University of Freiburg, Bertoldstr. 17, 79098 Freiburg, Germany; 4 Forest Health and Biodiversity, Natural Resources Institute Finland (Luke), Latokartanonkaari 9, 7, 00790 Helsinki, Finland

**Keywords:** Drought stress, Fungi–host relationship, Genotypic variation, Necrosis, Norway spruce, Root rot

## Abstract

Norway spruce *Picea abies* is one of Europe’s most economically important tree species. However, it is highly susceptible to the root rot fungus *Heterobasidion parviporum*. Climate change will benefit the pathogen as the tree host is weakened by, e.g., extended drought. Breeding can improve forest health, and several root rot genetic markers are suggested to improve the resistance of Norway spruce. This study aimed to compare the resistance (here defined as necrosis length) of Norway spruce families and genotypes against two strains of *H. parviporum* under different water availabilities. Our results show that the family and the genotype within the family have an impact on the necrosis length that is related to the aggressiveness of the fungal strains. Under low water conditions, the necrosis increased only in horizontal directions in phloem and sapwood. Similarly, the growth (seedling height) was not disturbed by abiotic stress (less water), indicating that the stress level (drought) was too low in this setting. The knowledge gained in this study could improve forest health in the changing climate by understanding the response of Norway spruce to pathogenic attacks under additional stress at the family level. This knowledge could be strategically used in forest breeding to improve the resistance of Norway spruce trees against root rot.

**Supplementary Information:**

The online version contains supplementary material available at 10.1007/s10342-023-01534-3.

## Introduction

Forest trees do not only have important roles in their economic and ecological value; they also mitigate the effects of climate change by storing carbon. However, fungal pathogens endanger the health of forests (Wingfield et al. 2015) and thus threaten these stocks. Norway spruce (*Picea abies*) is one of the highly valued tree species in Europe, but it is the main host of the pathogen *Heterobasidion parviporum* (Garbelotto and Gonthier 2013). Infections from this fungus make the trees highly susceptible to windthrow and lower their resistance, e.g., against bark beetles (Netherer et al. 2021). The continued growth of Norway spruce is threatened by root rot caused by members of the *Heterobasidion annosum *sensu lato species complex (Oliva et al. 2011; Piri 1996; Piri and Korhonen 2001, 2007). The economic loss due to this fungal complex was estimated to be ~ 800 million euros per year in Europe already 20 years ago (Asiegbu et al. 2005, Woodward et al. 1998), which should amount to about 1.2 billion euros as of 2020, given the present-day monetary value.

Diseased Norway spruce are difficult to detect, as the infected tree might not necessarily show any visible signs of symptoms (Allikmäe et al. 2017). Clearcutting is vastly creating new propagation sites for *H. parviporum*. The primary infection route is through basidiospores produced by the basidiocarp. These spores, carried by the wind, land on newly exposed surfaces, such as wounds on roots and stems or recently cut stumps (Garbelotto and Gonthier 2013; Redfern and Stenlid 1998; Swedjemark 1995). The fungus spreads to healthy trees via mycelium growth through the root network (Garbelotto and Gonthier 2013).

As the environment changes, there is an increasing possibility of extreme drought increasing in frequency and magnitude, posing even more significant threats to the forest (Allen et al. 2010, IPCC 2014, Senf and Seidl 2021). For example, in Germany, Norway spruce-dominated stands are particularly sensitive to drought (Bolte et al. 2021; Terhonen et al. 2019), and trees in Germany are declining due to extreme weather conditions, especially in areas with poor water supplies (BMEL 2021). For these reasons, research to test the environmental impact of this pathosystem can provide new strategies to limit the root rot disease. Selecting more adaptive and resistant trees through breeding can give solutions for this. Norway spruce has an inherent resistance to pathogenic attacks, including the *Heterobasidion* species. Several studies have proven that this intrinsic component plays a role in the susceptibility of Norway spruce to this pathogen.

The resistance of Norway spruce against *H. parviporum* is a quantitative trait (Arnerup et al. 2010, Capador‐Barreto et al. 2021, Lind et al. 2014). Lind et al. (2014) could map four quantitative trait loci in the *P. abies* genome for four distinct resistance traits against *H. parviporum*. These resistance traits included, e.g., lesion length at the inoculation site. For resistance breeding, inoculations trials in different families are needed to define if specific genotypes are suitable in (able to restrict the necrosis development) breeding programs. These traits are associated with several known defense responses with variation depending on the environment (Capador‐Barreto et al. 2021, Elfstrand et al. 2020; Yeoh et al. 2021). Therefore, understanding the family variation in tree response to pathogenic attacks under abiotic stress could aid in this process. Our objectives were to compare the variation in resistance to *H*. *parviporum* infection among Norway spruce families under abiotic stress, assess the effect of drought on seedling growth and estimate correlations between lesion size and genotypes.

## Materials and methods

### Plant materials

Plant material included 800 3-year-old Norway spruce (*P. abies*) seedlings received from the Haapastensyrjä field unit (60° 37′ 34.9″ N 24° 27′ 34.9″ E) of the Natural Resources Institute Finland (Luke). Materials consisted of eight families (ID: 38, 40, 41, 42, 43, 47, 48, 50) with 82 genotypes, each genotype having 3–10 ramets per clone. Seventy-three (73) genotypes had ten ramets, four genotypes had nine ramets, three had eight ramets, one had seven ramets, and one genotype had three ramets. The seedlings were established in the Forest Botany and Tree physiology greenhouse in Göttingen, Germany (51° 33′ 28.4″ N 9° 57′ 30.5″ E). Seedlings were planted (March 5th, 2020) in 3-L plastic pots filled with 2.5 L fertilized peat (Floragard, TKS®2 Instant Plus, PERLIGRAN® Extra 2–6 mm, Hermann Meyer KG, Rellingen, Germany). The potted seedlings received tap water to maintain moist soil.

### Fungal material

Two heterokaryotic *H. parviporum* strains, received from the strain collection of Natural Resources Institute Finland (collected by Dr. Tuula Piri), were used for the inoculation; *H*. *parviporum* strain 1 (Hpa 1 – strain number: SB 2005 9.16, isolated from a Norway spruce stump Solböle, Finland) and *H*. *parviporum* strain 2 (Hpa 2—strain number SB 2014 2.69, isolated from an infected Norway spruce seedling, Solböle, Finland) (Terhonen et al. 2022). The fungal isolates were plated on 1.5% Malt Extract Agar (MEA) and cultured in the growth chamber (Constant climate chamber Memmert HPP 750) for 2 weeks at 21 °C (in darkness) before inoculation.

### Experimental design

The experiment was run from July (July 22nd, 2020) until February (February 3rd, 2021). According to watering treatment, the seedlings were grouped into two (normal and low) groups of 400 plants per category. The plants received watering based on these groups, i.e., the seedlings in the 50% group received half the quantity of water received by the seedlings in the 100% class. The watering was adjusted according to the observed temperatures and soil moisture content (aiming for constantly moist soil for the 100% group). The soil moisture was measured before watering using a tensiometer—HH2 device equipped with the ML2x sensor (Delta-T Devices Ltd., Cambridge, UK). The temperature and humidity were recorded every watering day (Monday and Friday) from August until November with the digital thermometer. The experiment was carried out under ambient light conditions. The average monthly temperatures in the greenhouse were 31.8 °C (July), 25.9 °C (August), 23.2 °C (September), 15 °C (October) and 11.8 °C (November).

The normal water treatment group received 576 mL × 2 times per week, while the low water treatment received 288 mL × 2 times per week. The water amount was later adjusted to 384 mL/192 mL twice a week. The watering amount was further reduced to 192/96 mL (18.08.2020). The drought experiment lasted 16 weeks (22.07.2020–10.11.2020), and the seedlings were watered normally until the end of the experiment (03.02.2021). Due to Covid-19 restrictions, the watering treatment started in July 2020 instead of an anticipated earlier start of treatments.

### Inoculation

The bark of the seedlings was punched through with a sterile 5-mm cork borer to reach the sapwood surface; this was done at the height of ~ 10 cm from the base of the stem. The trees were artificially inoculated by placing equal-sized plugs of either *H. parviporum* strains (Hpa 1 and Hpa 2) or 1.5% malt extract agar (MEA) as mock control and sealed with Parafilm® (inoculations done on July 22, 2020) placing the fungal hyphae directly on the sapwood. Two hundred twenty-two (222) plants were inoculated with the first strain of *H. parviporum* and 222 with the second strain. Two hundred twenty-two (222) plants served as the mock control group inoculated with 1.5% MEA, while 134 plants remained non-treated. The plants in each inoculation group were divided into watering groups based on family numbers resulting in equal amounts in 50% and 100% water availability.

### Material collection and measurement

The growth data were measured after planting (diameter, starting height) and end of the watering experiment (final height, current year growth). The diameter was measured at ~ 5 cm from the stem base. Seedlings were harvested 28 weeks after inoculations; the stems were cut from ~ 10 cm above and below the inoculation point and stored at − 20 °C before measurements. The necrosis in the phloem was measured by gently scraping off the bark with a scalpel and measuring the surrounding lesion in both vertical and horizontal directions. The stem was then further scraped to assess the extent of damage at the sapwood level. The lesion length and width in phloem and sapwood were measured using a digital caliper.

### Statistical analysis

#### Growth

The soil moisture content between 50 and 100% was compared with t test at each sampling point. Data were analyzed using the SPSS version 28.0 (IBM Corporation, New York, NY, USA) and R (R Core Team 2021). Sixty-five (65) plants died during the experiment; one-way ANOVA (aov) was run to see if the death was due to a specific inoculation treatment. The remaining 735 plants were used for the growth analysis. One hundred twenty-three (123) out of 134 trees were analyzed as non-treated; 60 were grouped to normal water treatment and 63 to low water treatment. We constructed a generalized linear model to evaluate the non-treated seedling growth. The model included the water treatment group and family as initial fixed explanatory variables for the growth. Seven genotypes from these non-treated trees, each consisting of seven to ten ramets, were divided equally between different water treatments (each genotype ramets in low and normal water treatment). The growth of these 56 plants between two water treatments was further analyzed with a t test. Similarly, a generalized linear model was constructed to evaluate all seedling growth; this model included all the seedlings used in the experiment (*H. parviporum* strains 1 and 2, mock-inoculated and non-treated). This model had water treatment, inoculation treatment and family as initial fixed explanatory variables for the growth.

For the growth of plants, data distribution was assessed by means of the Shapiro–Wilk test in R (Royston 1982). Due to a deviation from normally distributed data, Levene’s test was used to test for homogeneity of variances. The Kruskal–Wallis test was done for homoscedastic data, and for heteroscedastic data, a Welch’s ANOVA was used. Lastly, when a dependent variable differed between treatments, post hoc analyses were performed using Dunn–Bonferroni for the analysis carried out with the Kruskal–Wallis’s test and Games–Howell multiple comparison test for the analysis carried out with Welch’s ANOVA test. Spearman’s correlation analysis was used to determine correlations between seedling growth (height_start_) and family. The impact and correlations were considered statistically significant if the *p* value was below the threshold of 0.01. The smaller *p* value was chosen to gain stronger evidence that a certain effect (family, inoculation, water availability, height_start_) was significant.

#### Necrosis

For the necrosis analysis, the non-treated plants (123) were excluded. A generalized linear model was also constructed to evaluate the fixed effects of the different treatments (mock-inoculated-control, *H. parviporum* strains 1 and 2) under different water treatments (high, low) on necrosis (length/width) in phloem and sapwood. Initial fixed explanatory variables in the necrosis length model included family, inoculation, water treatment, seedling growth and seedling start height.

The size of necrosis caused by *H. parviporum* strains (length/width) in phloem and sapwood per family (within genotype) was further evaluated. The data distribution was similar to the above assessed by employing the Shapiro–Wilk test in R. Due to a deviation from normally distributed data, Levene’s test was used to test for homogeneity of variances. The Kruskal–Wallis’s test was done for homoscedastic data, and for heteroscedastic data, a Welch’s ANOVA was used. Lastly, when a dependent variable differed between treatments, post hoc analyses were performed using Dunn–Bonferroni for the analysis carried out with the Kruskal–Wallis’s test and Games–Howell multiple comparison test for the analysis carried out with Welch’s ANOVA test. The Wald Chi-square test was used to assess the interactions between variables. Spearman’s correlation analysis was used to determine correlations of seedling growth (height_start_) with the sizes of necrotic lesions (phloem/sapwood and length/width) caused by *Heterobasidion* strains. In this study, correlations were considered statistically significant if the *p* value was below the threshold of 0.01. Same as above, the smaller p value was chosen to gain stronger evidence that certain effect (family, genotype, inoculation, water availability, height_start_) has importance on necrosis size.

## Results

### Growth analysis

The soil moisture was statistically different (*p* = 2.79e−06) from the fifth week (Fig S1 supplementary data). The death of trees was not affected by the different treatments (*p* = 0.948). The death of seedlings was assumed to be random (the death of each seedling was observed during the experiment, not at the end of the experiment), and they were not used in data analysis.

**Fig. 1:**
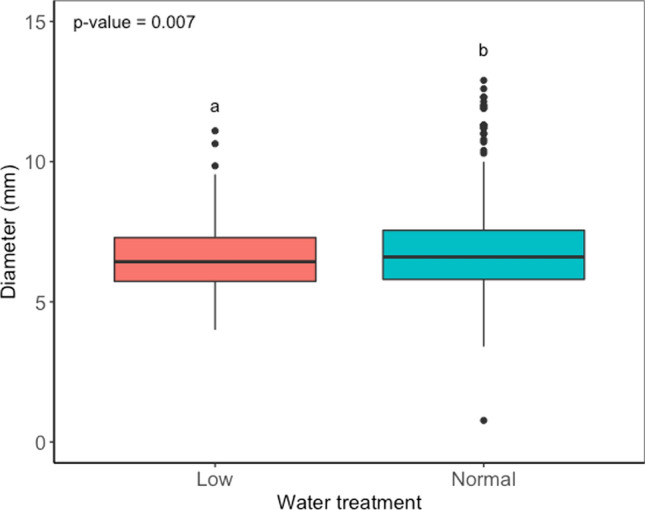
Effects of water treatment on plant diametric growth. Different letters above plots denote significantly different groups after post hoc test

**Fig. 2:**
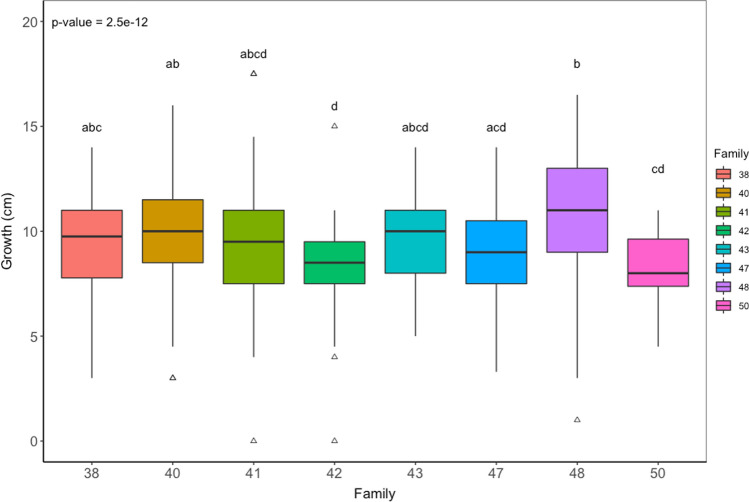
Effects of Norway spruce family on the height growth (median height growth for each family). Different letters above plots denote significantly different groups after post hoc test

The height growth between non-treated ramets (56 plants) was not statistically different (*p* = 0.875) between the water treatments. Furthermore, the growth model for all non-treated plants (123 trees) showed that the water treatment did not impact the height (*p* = 0.665). Similarly, for all trees in the experiment, the growth model showed that the water treatment did not impact height growth (*p* = 0.974). However, a significant effect of water treatment was observed in the diametric growth (*p* = 0.007) (Fig. 1). Further differences observed were due to the family (*p* < 0.001). The different treatments (*H. parviporum* 1 or 2, mock control or non-treated) did not impact the growth (*p* = 0.526). The diameter, family and genotypes were the fixed explanatory for all trees for the differences between growth (*p* < 0.001). Welch’s analysis of variance showed a significant difference in height growth among the different families (Fig. 2) and genotypes (Fig S2 supplementary data). Games–Howell test revealed that plants of family 48 grew significantly taller than families 42 (*p* < 0.001), 47 (*p* < 0.001) and 50 (*p* < 0.001). Family 42 grew statistically less compared to families 38 (*p* = 0.009), 40 (*p* = 6.78e−07) and family 48 (*p* = 6.5e−10). Similarly, the variation in the diameter of plants was statistically significant in relation to the families (*p* = 5.01e−13, Fig S3 supplementary data). Family 43 had the lowest diameter statistically compared to families 38, 40, 41, 42 and 47.

### Necrosis analysis

**Fig. 3:**
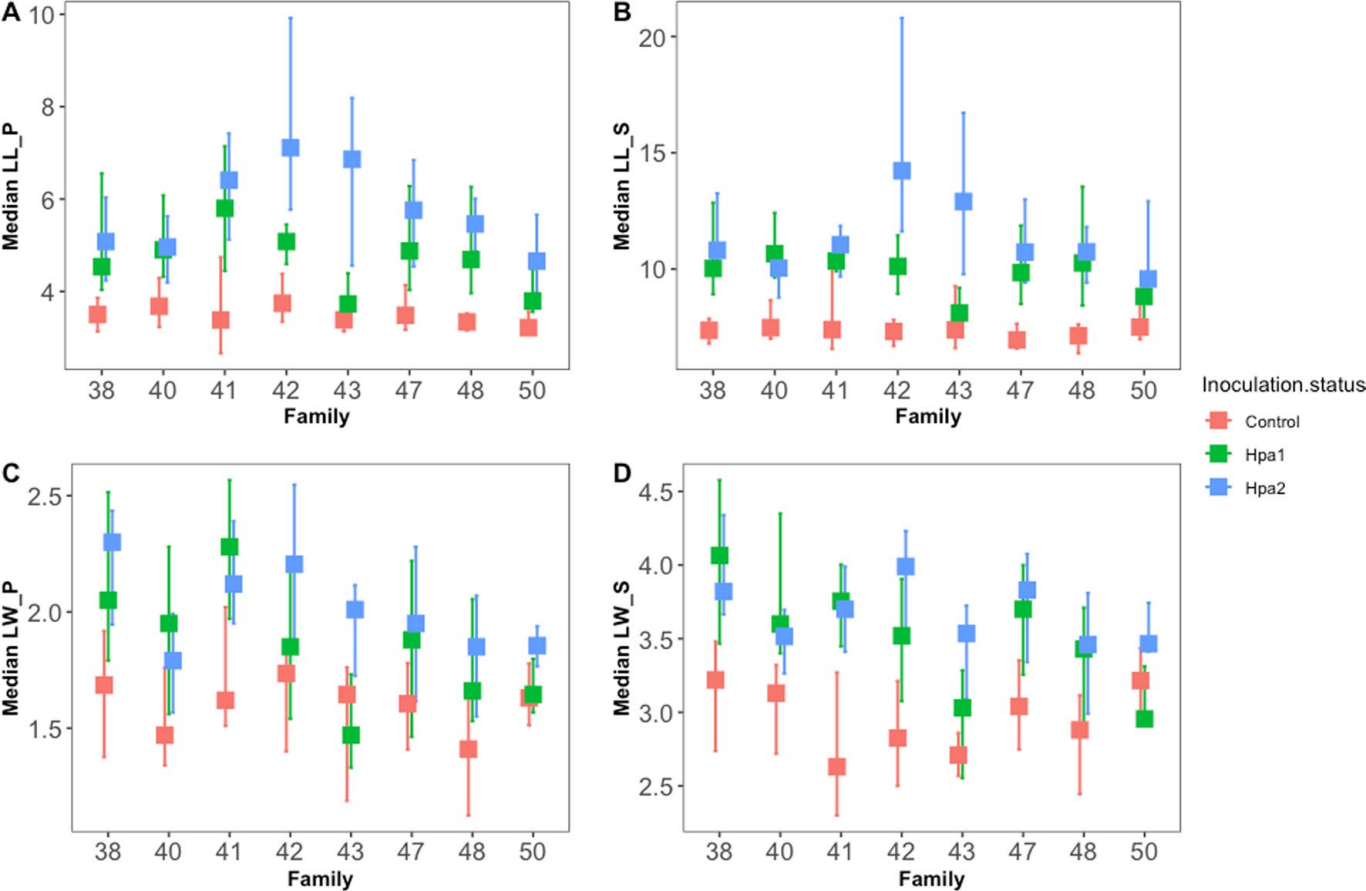
Interaction between families and different inoculation treatments (median values) **a** *LL_P* lesion length in phloem, **b** *LL_S* lesion length in sapwood, **c** *LW_P* lesion width in phloem **d** *LW_S* lesion width in sapwood

**Fig. 4:**
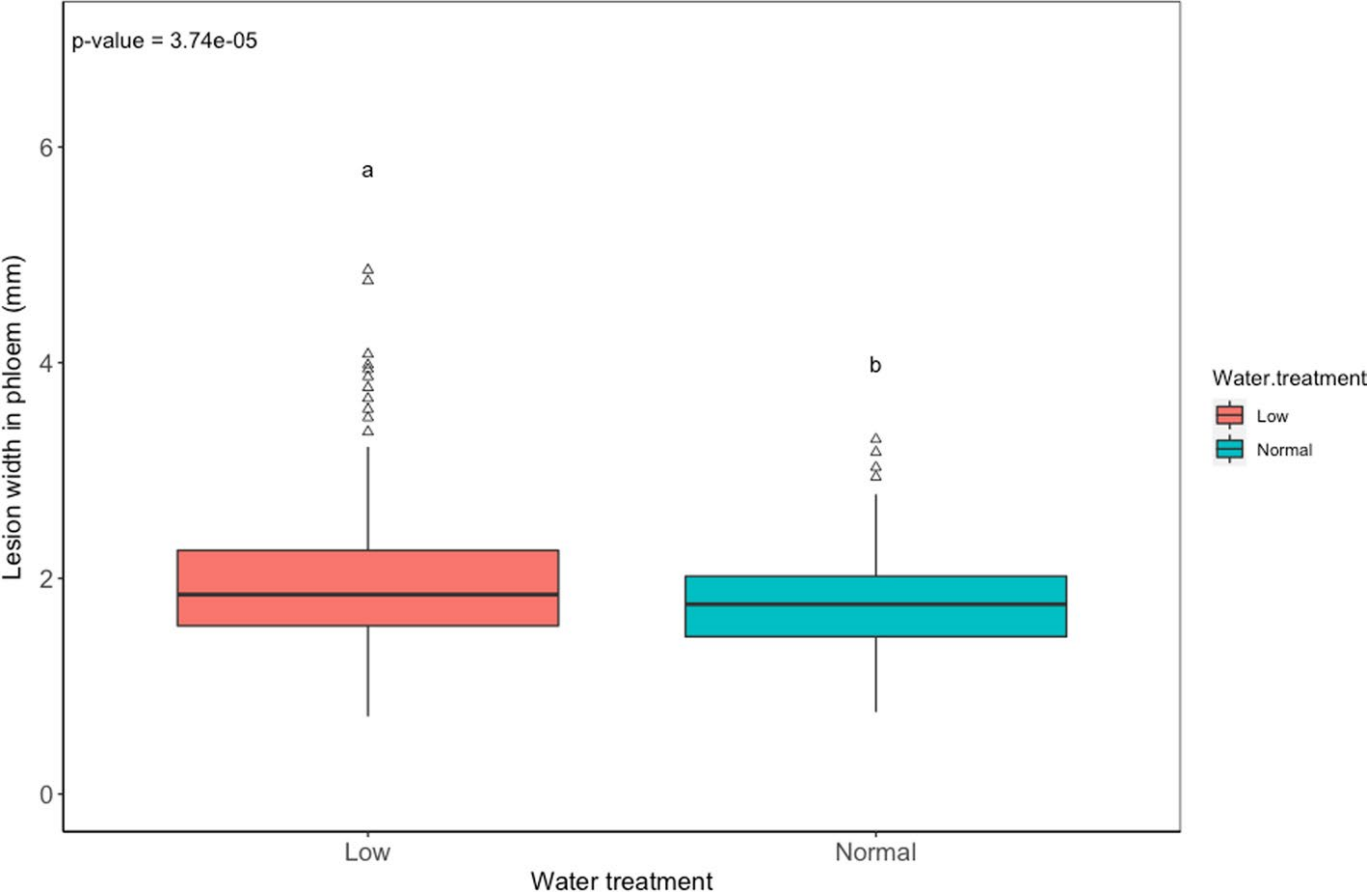
Impact of water treatment on lesion width in the phloem. Different letters above plots denote significantly different groups after post hoc test

The necroses in phloem and sapwood (length/width) were statistically different (*p* < 0.001) due to the family and different treatments (Table 1, Fig. 3). The lesion width was also influenced by starting height (Table 1). Necrotic lesions (all treatments together) in the phloem were significantly wider (*p* < 0.01) in the horizontal direction for the low water treated plants when compared to the optimally watered plants (Fig. 4). The analysis of the impact of water treatment on lesion size shows no significant differences in lesion length (Table 1).

**Table 1:** A generalized linear model for response variables. Models included interactions between the dependent and response variables

	Variable	Fixed effect	*p* value
Sapwood	Necrosis, length	Family	> 0.01
		Water	0.053
		Inoculation	< 0.01
		Starting height	0.169
		Growth_height_	0.958
	Necrosis, width	Family	< 0.01
		Water	0.098
		Inoculation	< 0.01
		Starting height	< 0.01
		Growth_height_	0.015
Phloem	Necrosis, length	Family	< 0.01
		Water	0.169
		Inoculation	< 0.01
		Starting height	0.366
		Growth_height_	0.29
	Necrosis, width	Family	< 0.01
		Water	< 0.01
		Inoculation	< 0.01
		Starting height	< 0.01
		Growth_height_	0.419

**Fig. 5:**
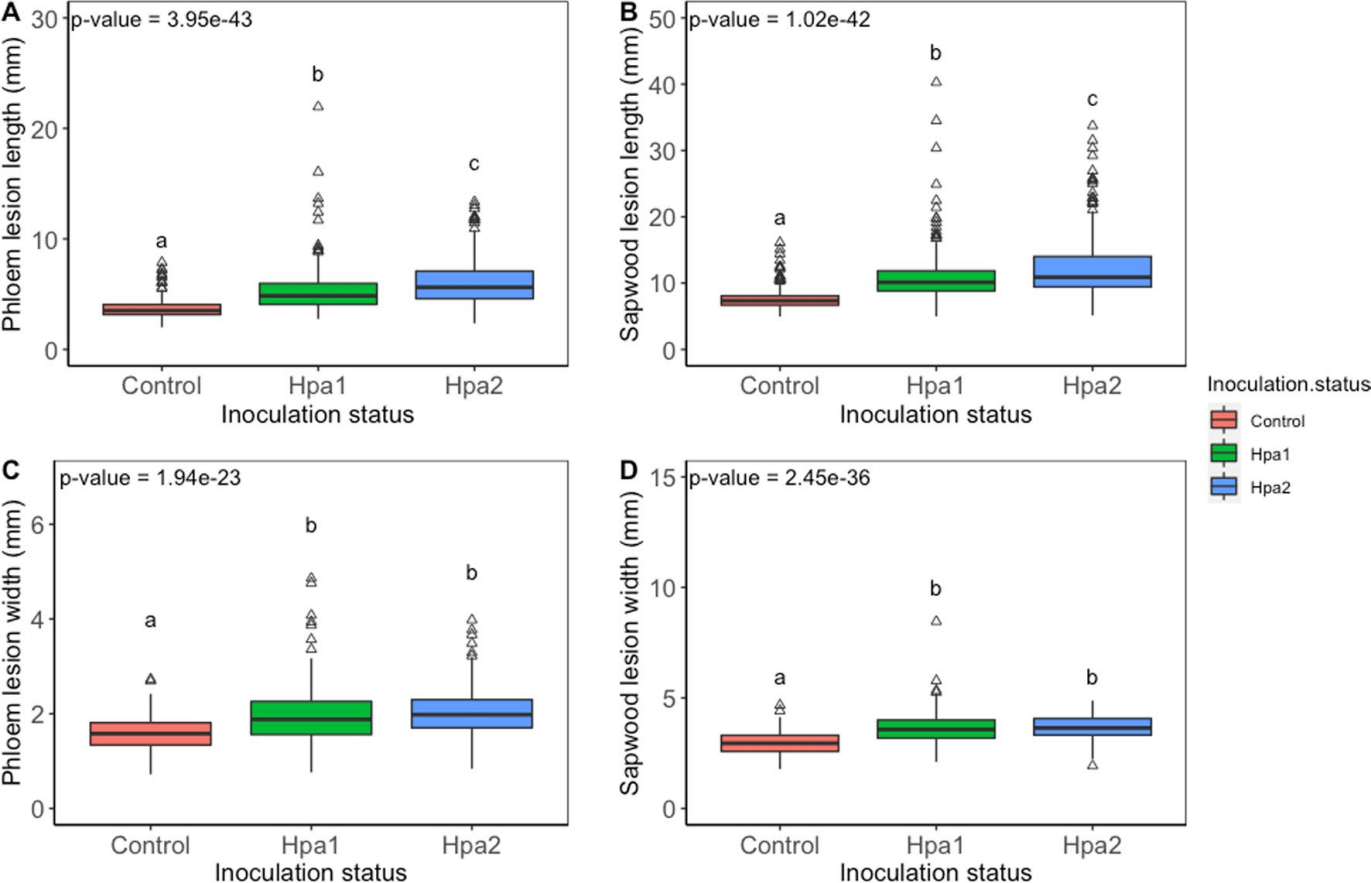
Inoculation effect on lesion size. **a** Lesion length in phloem, **b** lesion length in sapwood, **c** lesion width in phloem, **d** lesion width in sapwood

The lesion length and width in the sapwood are larger than those in the phloem, and *H. parviporum* strains caused more necrosis than in the mock control. The lesion lengths in the phloem and sapwood were significantly different between the strains (Fig. 5a, b).

The strain Hpa 2 caused statistically higher necrosis (lesion length) in both phloem and sapwood than the other strain (Hpa 1) (*p* = 0.002). The only significant difference in the lesion width for phloem and sapwood was between the *Heterobasidion* inoculated and the mock control plants (Fig. 5). The lesion width is not statistically different between *H. parviporum* strains (Fig. 5c, d).

There were significant differences in lesion length and width in the phloem among genotypes (Supplementary data; Table 2). In the sapwood, there were significant differences in the lesion length (*p* = 0.001) and width (*p* = 1.9e−04) among genotypes (Supplementary data; Fig S4).

### Correlation analysis

**Fig. 6:**
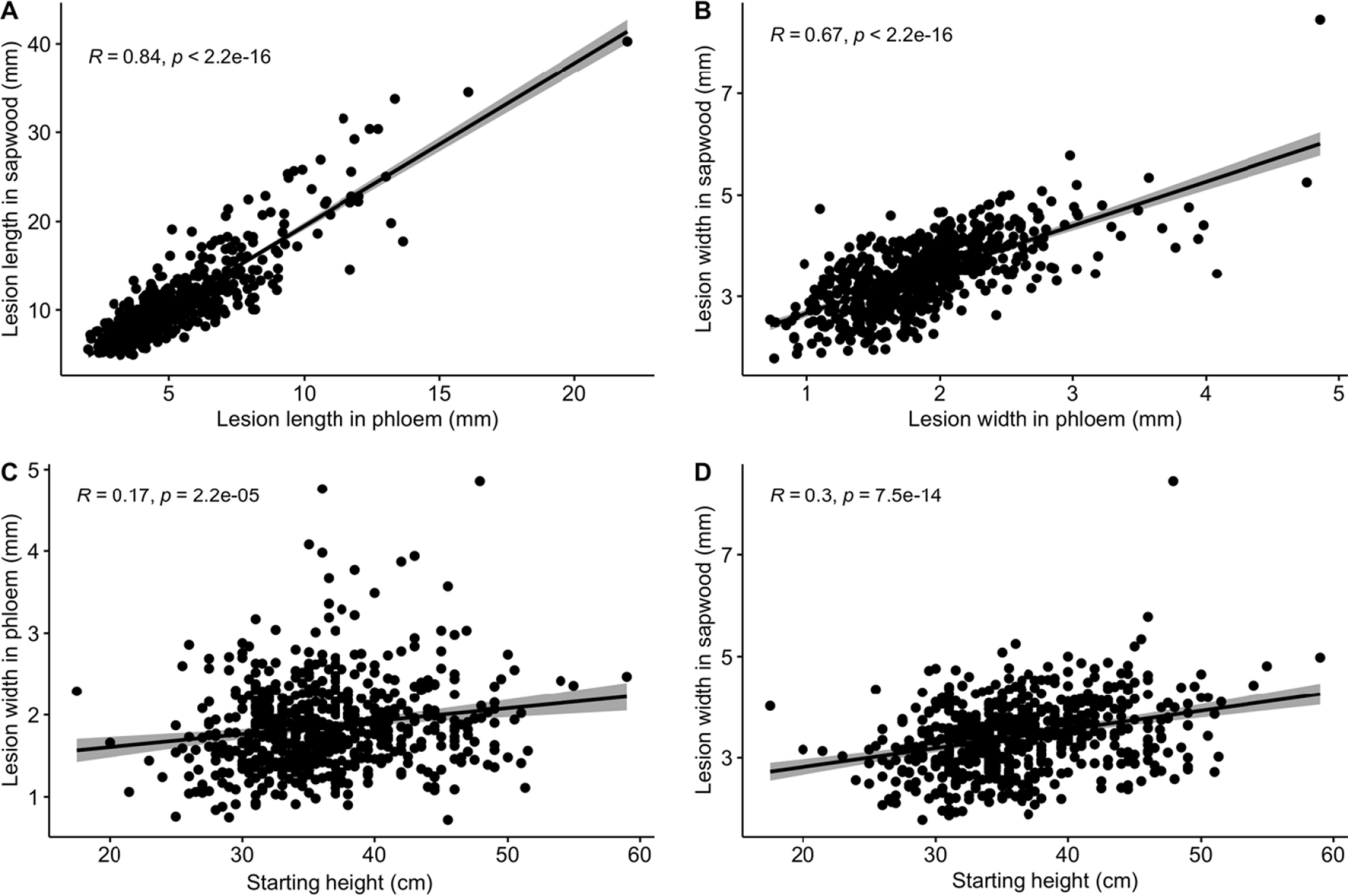
Correlation analysis between** a** lesion length in phloem and sapwood, **b** lesion width in phloem and sapwood, **c** lesion width in phloem and starting height, **d** lesion width in sapwood and starting height

**Fig. 7:**
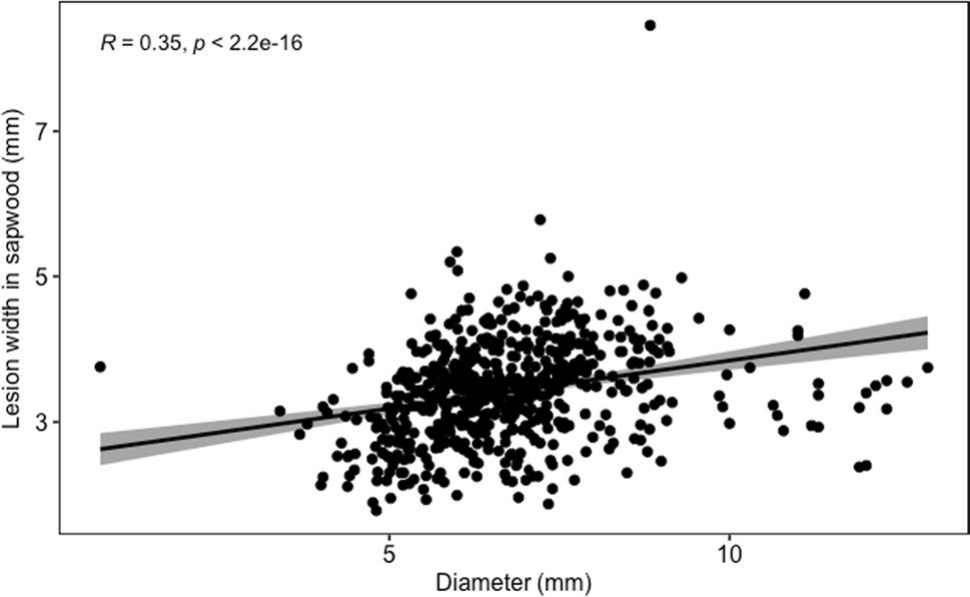
Correlation analysis between stem diameter and lesion width in the sapwood

There is a strong positive correlation between the lesion sizes in both phloem and sapwood (*p* < 0.01) (Fig. 6a, b). The higher the lesion in the phloem, the higher it is in the sapwood. There were no significant correlations between lesion length and starting height, but there are significant positive correlations between the lesion width and starting height (Fig. 6c, d). Correlations between diameter and lesion width in sapwood also resulted in a significant moderate positive correlation (*R*; 0.35, *p* < 001) (Fig. 7). A negative correlation was revealed between growth (height) and lesion length in the phloem (*R* = − 0.096, *p* = 0.016) and the sapwood (*R* = − 0.048, *p* = 0.23) (Fig S5 supplementary data).

### Discussion

#### Growth

In this study, the height growth of Norway spruce seedlings was not affected by water availability; rather, the differences were due to the stem diameter, families and genotypes. Water stress impedes plant growth (Hsiao 1973; Bigler et al. 2006, Rötzer 2017), and understanding the response of trees to drought is important for predicting how they will react to a changing climate (McDowell 2011). Taeger et al. (2013) investigated the effects of drought on two different populations of *Pinus sylvestris* seedlings (Germany and Spain) in a greenhouse experiment. Overall, drought led to reductions in growth, while differences were observed between populations (Taeger et al. 2013). Similarly, higher drought reduced the growth of *Pinus halepensis* pine in the semi-arid forest (Klein et al. 2014). Limited water availability can negatively influence Norway spruce seedling growth (Linnakoski et al. 2017; Terhonen et al. 2019). In contrast to our expectations, the height growth of the seedlings was not significantly impacted by the different treatments (water treatment nor inoculation) but by the genetic variation.

Terhonen et al. (2019) could show that 50% of water availability was enough to disturb the growth of 3-year-old Norway spruce seedlings in a 15-week interval. However, in our setting (due to COVID-19 restrictions), we started the water treatment rather late (in late July) compared to Terhonen et al. (2019) (early April). The abiotic stress was not critical anymore for the Norway spruce growth after this time (even though the terminal growth period was still ongoing). The height growth most likely had ended when the watering experiment started and cannot be considered a marker for stress level in this study. Although the height growth was not impacted by low water availability, the diametric growth was. Host–pathogen interaction, however, may have been affected by the drought treatment as lesion width in phloem was larger in low-watered plants compared to optimally watered plants. As a result, plants that received optimum watering grew better (larger in diameter) than those that received low watering.

Similarly, Rötzer et al. (2017) found that drought significantly decreases the diameter increment of Norway spruce. Drought stress has also been reported to negatively impact the tree ring width of some Norway spruce clones as a result of a reduction in the cambial activity (Gryc et al. 2012). Due to limited water availability, cells necessary for the formation of a new ring are formed in fewer quantities in comparison with other clones that were not subjected to water stress (Gryc et al. 2012).

However, it was clear that the growth also varied between families and genotypes. This is in line with several studies showing differences in the growth of Norway spruce clones/genotypes (Hannerz et al. 1999; Jansone et al. 2020). Liu et al. (2022) could also show significant growth differences among 3-year-old Norway spruce clones inoculated with *H*. *parviporum*.

Similarly, in a study carried out on Norway spruce plantation, clonal and genotypic differences were evident in the growth rate of the Norway spruce (Zeltiņš et al. 2022). Additionally, Chen et al. (2018) found that Norway spruce genotypes in a Swedish breeding program differed widely in growth traits. Besides the variation in height (growth), the genotypes also differed in diameter. This is in line with the study by Zeltinš et al. (2022), where the diameter growth differed significantly among the genotypes.

### Necrosis

Here, we show resistance (necrosis length) phenotyping results based on 3-year-old *Picea abies* families originating from controlled crosses between plus trees of Norway spruce, Finland. Necrosis length was significantly different among Norway spruce genotypes*.* This is consistent with results showing variability in Norway spruce reaction to *H*. *parviporum* between genotypes (Swedjemark 1995; Skrøppa et al. 2015). Although there was no impact of water treatment on plant height, there was a significant difference in lesion width in the drought-treated plants compared to the optimally watered plants. The lesion width has been shown to increase under drought in Norway spruce after inoculation with *H*. *annosum* s.s. (Terhonen et al. 2019). At the same time, inoculation with *H*. *parviporum* increased lesion length in drought-treated plants compared to normally watered plants (Terhonen et al. 2019). Madmony et al. (2018) inoculated 2-year-old branches of different Norway spruce clonal ramets (4-year-old) with *H*. *parviporum* under well-watered and drought environments, which led to increased pathogen growth in well-watered seedlings. In our experiment, the water treatment did not have an impact on height growth and necrosis; the effect of the water treatment was only shown in the diametric growth and lesion width in the phloem. Controversial results have been found as necrosis length has been shown to increase due to lower water availability (Terhonen et al. 2019). Reasons for this might be the different experimental settings, as both genotype and environment can impact the defense (Potts and Hunter 2021). It is evident that the resistance (necrosis width/length) varies between families and genotypes.

In our study, the death of trees was considered random. Pathogenic fungi induce defensive responses in trees, which aim to restrict the growth of the pathogen and foster recovery (Berryman 1972). This defense uses a lot of the host resources. Therefore, plant growth can be negatively impacted by allocating resources to defense instead (Walters and Heil 2007). Several studies have reported negative (Mukrimin et al. 2018; Liu et al. 2022), positive (Karlsson et al. 2008; Chen et al. 2018; Mukrimin et al. 2019) and negligible correlations (Lamara et al. 2018; Camisón et al. 2019) between defense and growth in different tree species. The results of our study did not support this hypothesis between growth and resistance (lesion length) in phloem and sapwood of Norway spruce because there was no correlation, although the values are very close to a weak negative correlation. Seedlings’ growth was not affected by lesion length in phloem and sapwood.

Similarly, Steffenrem et al. (2016) did not find a genetic correlation between growth and resistance. However, a significant positive correlation was found between lesion width and seedlings’ starting height. The same observations have been made before (Karlsson et al. 2008; Terhonen et al. 2019). This indicates (the higher starting height equals higher lesion width) that seedlings with higher values of growth parameters (in this case, taller seedlings) are not unquestionably less sensitive to *Heterobasidion* inoculation.

Studies have focused on the defense response of *Picea abies* to different pathogens (Skrøppa et al. 2015; Terhonen et al. 2019; Axelsson et al. 2020). They have shown differences in the defense response of Norway spruce clones/genotypes to fungal infection. Still, little focus has been placed on the degree of virulence between different strains of the same pathogen. Axelsson et al. (2020) showed variabilities in defense response among Norway spruce clones after inoculation with *Endoconidiophora polonica* and *Heterobasidion parviporum*. Inoculation with *H*. *parviporum* strongly induced terpenes in all clones as a response to defense from pathogenic attack, but the quantity of terpenes induced varied among clones. One of the important results of our study is that the virulence (lesion length) varied between the fungal strains of *Heterobasidion* used. *H*. *parviporum* strain 2 showed to be more pathogenic (caused longer necrosis) than the first strain. This could be due to the isolation source (stump versus seedling), as there may be a trade-off between saprobic and pathogenic competence (Olson et al. 2012). Similarly, Linnakoski et al. (2017) showed that the virulence of *Endoconidiophora polonica* varied among the different strains. The differences observed in the virulence (necrosis length) among pathogenic strains in this and previous studies highlight the importance of using more than one strain in inoculation studies.

Tree breeding has been shown to increase the growth performance of forest trees (Jansson et al. 2017), particularly Norway spruce, by 8–20%. From our study, considering the reaction of plant hosts to different strains is essential for selecting resistant genotypes to be used in reforestation and breeding, as there could be variations in resistance to various strains.

Climate change is expected to cause warmer temperatures and drought (IPCC 2014), which will have unpredicted effects on pathogen behavior, hosts’ vitality and their ability to fight infection. As a result, there are several risks facing future generations of Norway spruce forests. Further research is needed with stricter water stress taking different genotypes and fungal strains into account to assess the physiological and genetic factors influencing host resistance and pathogen virulence in the face of climate change. Results from this study contribute to the body of research on host resistance, and the plant–pathogen interactions can serve as a foundation for further research.

## Conclusion

This study highlights that the genotypes of Norway spruce have an impact on the growth and response to artificial infections for different *H. parviporum* strains. Therefore, further research is suggested to assess Norway spruce susceptibility against several pathogenic strains. The transcriptomics studies for these genotypes could reveal traits that control lesion length at the inoculation site. These could be used to find new molecular markers for resistance breeding and will be the focus of our future studies.

## Supplementary Information

Below is the link to the electronic supplementary material.

Supplementary file1 (DOCX 593 KB)

Supplementary file2 (DOCX 22 KB)
