# Supplementary material for: Genetic variation of Picea abies in response to the artificial inoculation of Heterobasidion parviporum
Source: Eur J For Res. 2023 Jan 27;142(2):443–53. doi: 10.1007/s10342-023-01534-3 (PMC9880357; doi:10.1007/s10342-023-01534-3)
Supplement: Supplementary file 1 — Supplementary file1 (DOCX 593 KB) [file 10342_2023_1534_MOESM1_ESM.docx]

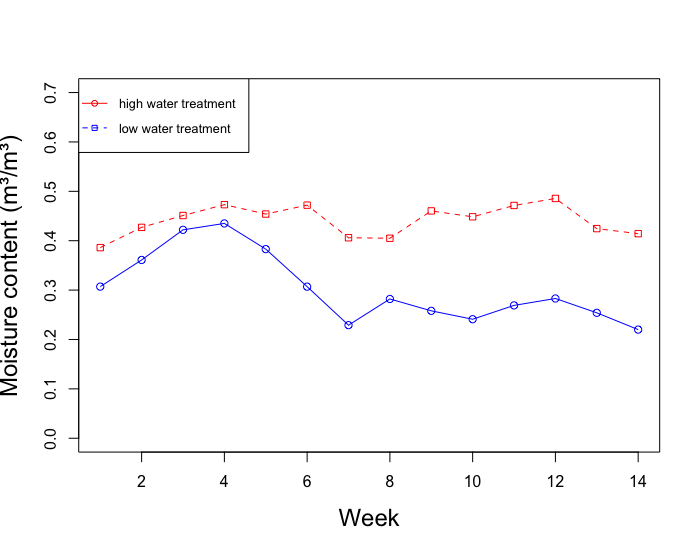


**Fig S1 Soil moisture content between low and well-watered plants (significance level;** *p* = 2.79e-06**).** There are fourteen weeks because there were no taken for two weeks in the middle of the experiment


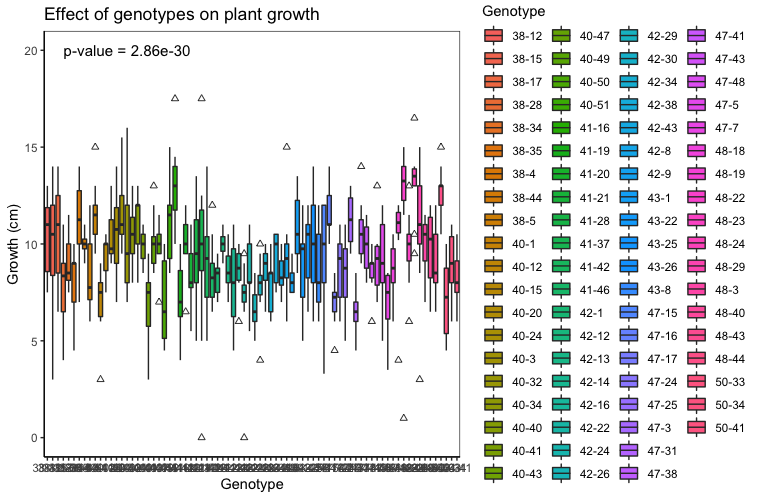


**Fig S2 Effects of Norway spruce genotypes on plant growth**


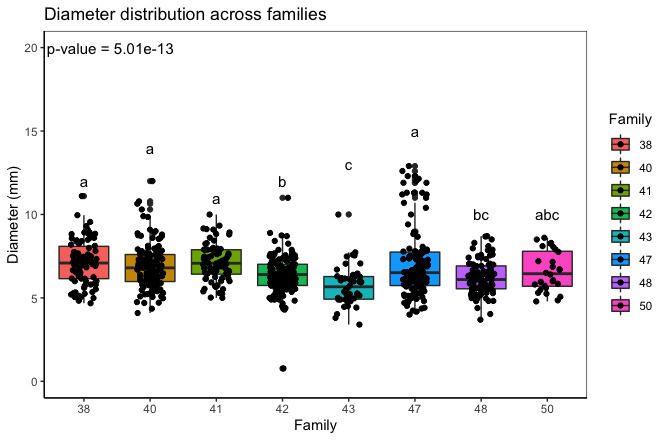


**Fig S3 Diameter distribution among families** (median diameter for each family. Different letters above plots denote significantly different groups after posthoc test)

**
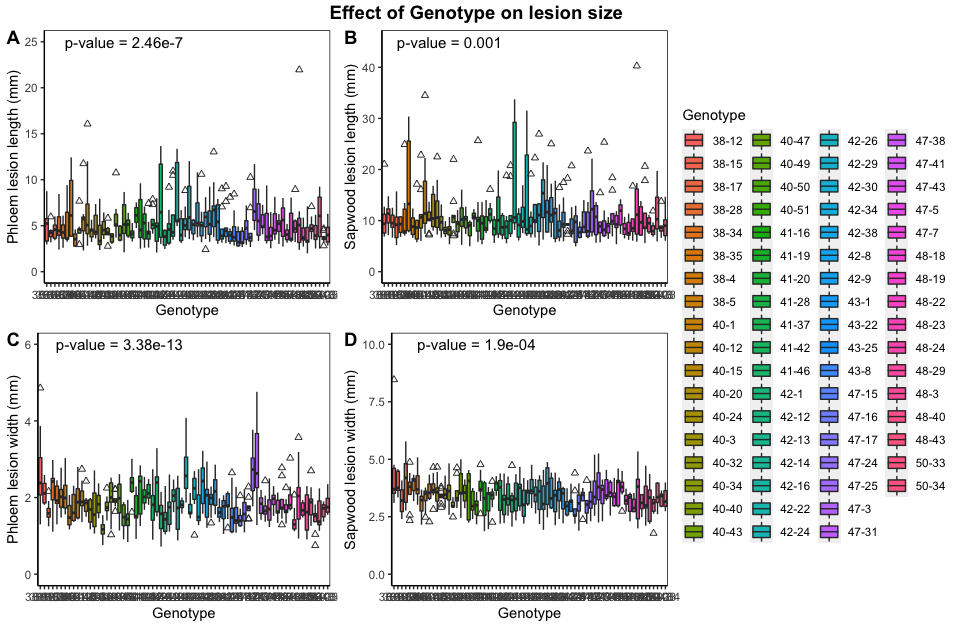
**

**Fig S4 Genotype effect on lesion size**. **a)** lesion length in phloem **b)** lesion length in sapwood **c)** lesion width in phloem **d)** lesion width in sapwood

**
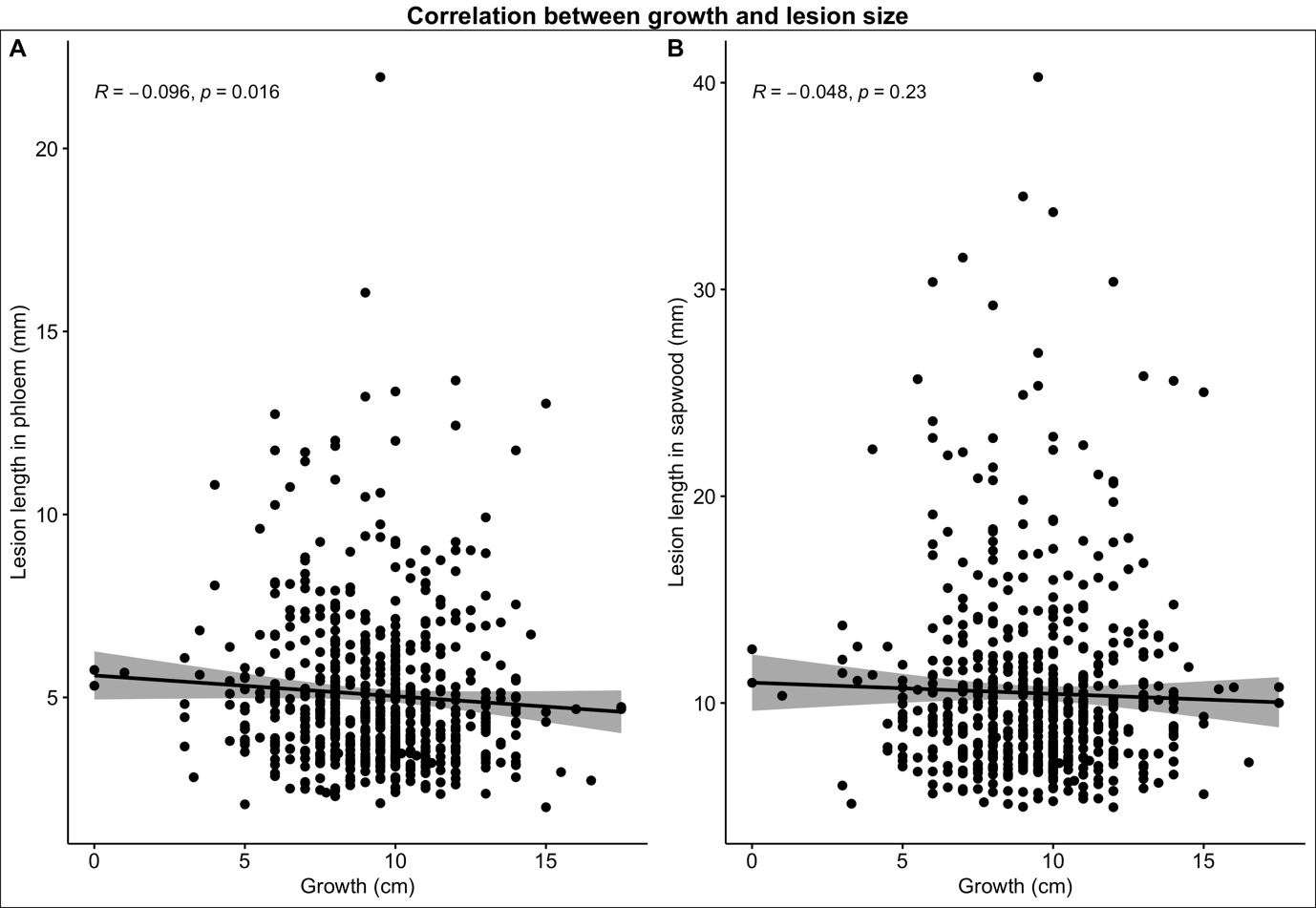
**

**Fig S5 Correlation analysis between growth and lesion length in a)** phloem, **b)** sapwood
