# Supplementary material for: Genetic variation of Picea abies in response to the artificial inoculation of Heterobasidion parviporum
Source: Eur J For Res. 2023 Jan 27;142(2):443–53. doi: 10.1007/s10342-023-01534-3 (PMC9880357; doi:10.1007/s10342-023-01534-3)
Supplement: Supplementary file 2 — Supplementary file2 (DOCX 22 KB) [file 10342_2023_1534_MOESM2_ESM.docx]

Table 1: Significant differences in lesion in phloem and sapwood among families. LL_P denotes lesion length in Phloem, LW_P denotes lesion width in the phloem, LW_S denotes lesion width in sapwood

| **.y.** | **group1** | **group2** | **n1** | **n2** | **statistic** | **p** | **p.adj** | **p.adj.signif** |
| --- | --- | --- | --- | --- | --- | --- | --- | --- |
| LL_P | 42 | 50 | 117 | 18 | -3.41117522645604 | 0.000646835082042906 | 0.0181113822972014 | * |
| LL_P | 42 | 43 | 117 | 36 | -3.13058744425898 | 0.00174457052102484 | 0.0488479745886954 | * |
| LW_P | 41 | 48 | 62 | 79 | -4.64871939945031 | 3.34002222311134E-06 | 9.35206222471175E-05 | **** |
| LW_P | 38 | 48 | 71 | 79 | -4.2810701998121 | 1.85996668309342E-05 | 0.000520790671266158 | *** |
| LW_P | 40 | 41 | 122 | 62 | 3.98438112342891 | 6.7656214845916E-05 | 0.00189437401568565 | ** |
| LW_P | 38 | 40 | 71 | 122 | -3.5692981213 | 0.000357938921086483 | 0.0100222897904215 | * |
| LW_P | 41 | 43 | 62 | 36 | -3.53088967739828 | 0.000414164453295332 | 0.0115966046922693 | * |
| LW_P | 42 | 48 | 117 | 79 | -3.4114812541816 | 0.000646109438076217 | 0.0180910642661341 | * |
| LW_P | 38 | 43 | 71 | 36 | -3.1828205049313 | 0.00145847998306918 | 0.0408374395259371 | * |
| LW_S | 38 | 43 | 71 | 36 | -4.78451891157609 | 1.71397360125504E-06 | 4.79912608351412E-05 | **** |
| LW_S | 38 | 48 | 71 | 79 | -4.59826885197763 | 4.26016018909622E-06 | 0.000119284485294694 | *** |
| LW_S | 43 | 47 | 36 | 107 | 3.27269117149149 | 0.00106528804654984 | 0.0298280653033955 | * |

Table 2: Significant differences in lesion in phloem and sapwood among genotypes. LL_P denotes lesion length in Phloem, LL_S denotes lesion length in sapwood, and LW_P denotes lesion width in the phloem

| **.y.** | **group1** | **group2** | **estimate** | **conf.low** | **conf.high** | **p.adj** | **p.adj.signif** |
| --- | --- | --- | --- | --- | --- | --- | --- |
| LL_P | 40-40 | 41-16 | 2.38333333333333 | 0.40441813167361 | 4.36224853499306 | 0.013 | * |
| LL_P | 41-16 | 47-15 | -2.51444444444444 | -4.76795261271894 | -0.260936276169949 | 0.019 | * |
| LL_P | 41-16 | 47-16 | -2.49541666666667 | -4.75194785384373 | -0.238885479489606 | 0.021 | * |
| LL_P | 41-16 | 50-33 | -2.38555555555556 | -4.40143557178511 | -0.369675539326002 | 0.013 | * |
| LL_P | 38-5 | 41-16 | 2.54444444444444 | 0.445984322771353 | 4.64290456611754 | 0.009 | ** |
| LL_S | 40-34 | 41-16 | 2.55222222222222 | 0.197250041100992 | 4.90719440334345 | 0.025 | * |
| LW_P | 38-15 | 48-22 | -0.75 | -1.49533759243925 | -0.00466240756075009 | 0.048 | * |
| LW_P | 38-34 | 40-34 | -0.808888888888889 | -1.55428420526788 | -0.0634935725098975 | 0.026 | * |
| LW_P | 38-35 | 40-34 | -0.845555555555555 | -1.65744196114143 | -0.0336691499696823 | 0.037 | * |
| LW_P | 40-20 | 40-34 | -0.675555555555556 | -1.28275735576863 | -0.068353755342481 | 0.02 | * |
| LW_P | 40-34 | 40-40 | 0.603333333333333 | 0.0206863388794454 | 1.18598032778722 | 0.037 | * |
| LW_P | 40-34 | 42-8 | 0.737777777777778 | 0.0537784006720795 | 1.42177715488348 | 0.027 | * |
| LW_P | 40-34 | 47-25 | 1.49111111111111 | 0.184120773319692 | 2.79810144890253 | 0.02 | * |
| LW_P | 40-34 | 47-31 | 0.634444444444445 | 0.0352644615719299 | 1.23362442731696 | 0.031 | * |
| LW_P | 40-34 | 48-24 | 0.511222222222222 | 0.00504410213066753 | 1.01740034231378 | 0.046 | * |
| LW_P | 40-34 | 50-34 | 0.666666666666667 | 0.0527267863195551 | 1.28060654701378 | 0.025 | * |
| LW_P | 40-50 | 41-16 | 0.858888888888889 | 0.00264135849415015 | 1.71513641928363 | 0.049 | * |
| LW_P | 40-51 | 41-16 | 0.971111111111111 | 0.10086609268272 | 1.8413561295395 | 0.019 | * |
| LW_P | 40-51 | 41-37 | 0.764444444444444 | 0.0415034049420123 | 1.48738548394688 | 0.032 | * |
| LW_P | 41-16 | 47-16 | -0.881944444444444 | -1.71062003163479 | -0.0532688572541017 | 0.03 | * |
| LW_P | 41-16 | 48-22 | -0.954444444444444 | -1.79029306518129 | -0.118595823707595 | 0.016 | * |
| LW_P | 41-16 | 48-40 | -0.947777777777778 | -1.79482685840363 | -0.100728697151925 | 0.019 | * |
| LW_P | 41-37 | 43-1 | -0.522222222222222 | -1.03292241613538 | -0.0115220283090601 | 0.042 | * |
| LW_P | 41-37 | 47-16 | -0.675277777777778 | -1.33031688267533 | -0.0202386728802275 | 0.04 | * |
| LW_P | 41-37 | 48-22 | -0.747777777777778 | -1.41373498273514 | -0.0818205728204136 | 0.019 | * |
| LW_P | 41-37 | 48-40 | -0.741111111111111 | -1.42625113421544 | -0.055971088006785 | 0.026 | * |
| LW_P | 40-34 | 41-28 | 0.86 | 0.252312089847911 | 1.46768791015209 | 0.002 | ** |
| LW_P | 40-34 | 42-22 | 0.624444444444445 | 0.140777545783701 | 1.10811134310519 | 0.006 | ** |
| LW_P | 40-34 | 47-43 | 0.743333333333333 | 0.141142582999115 | 1.34552408366755 | 0.007 | ** |
| LW_P | 38-15 | 40-34 | -0.974444444444444 | -1.64308088866347 | -0.305808000225418 | 0.001 | *** |
| LW_P | 40-34 | 40-47 | 0.76 | 0.248895989718388 | 1.27110401028161 | 0.001 | *** |
| LW_P | 40-34 | 41-16 | 1.17888888888889 | 0.397777487078828 | 1.96000029069895 | 0.001 | *** |
| LW_P | 40-34 | 41-37 | 0.972222222222222 | 0.415218434666576 | 1.52922600977787 | 0.000127 | *** |
| LW_P | 40-34 | 42-14 | 0.865555555555556 | 0.271742446560321 | 1.45936866455079 | 0.001 | *** |
